# Severe Pernicious Anemia: A Case Report

**DOI:** 10.7759/cureus.85088

**Published:** 2025-05-30

**Authors:** Leonor Simões Raposo, Andreia Pacheco, Maria Mendes

**Affiliations:** 1 General Practice, Family Unit Conde de Oeiras, Western Lisbon Local Health Unit, Lisbon, PRT; 2 Family Medicine, Family Unit Conde de Oeiras, Western Lisbon Local Health Unit, Lisbon, PRT

**Keywords:** intravascular hemolysis, macrocytic anemia, non-specific symptoms, pancytopenia, pernicious anemia, primary care role, vitamin b12 deficiency

## Abstract

Pernicious anemia is an infrequent condition and a recognized cause of vitamin B12 deficiency. It is an autoimmune disease that impairs the absorption of vitamin B12, leading to a significant reduction in its uptake. Clinical manifestations are often subtle and non-specific, yet potentially debilitating, which may delay diagnosis. However, with early recognition in primary care and appropriate replacement therapy, patients can recover their quality of life. Fatigue and asthenia are common complaints in primary care, highlighting the importance of careful clinical evaluation in the presence of nonspecific symptoms. Timely laboratory investigations are essential for the early detection of the disease and the prevention of serious complications.

## Introduction

Pernicious anemia is a common cause of vitamin B12 deficiency [1,2]. It is more common in females (2.7%) than males (1.4%) and is equally likely in Black and White females [3]. Pernicious anemia is an autoimmune condition that prevents the formation of the vitamin B12-intrinsic factor complex, which in turn decreases vitamin B12 absorption [3]. Generally, it takes about 10 years to clinically develop symptoms, so pernicious anemia may onset with subclinical vitamin B12 deficiency [2]. Pernicious anemia is often underdiagnosed, especially in younger individuals and non-white populations. Studies indicate that this condition may present at earlier ages in these groups, which can contribute to delayed or incorrect diagnoses [4]. Clinically, it manifests in a subtle and nonspecific manner, with asthenia and fatigue. It may present more specific manifestations such as glossitis (including pain, swelling, tenderness, and loss of papillae and/or hyperpigmentation of the tongue), neuropsychiatric manifestations such as symmetric paresthesias or numbness (affecting the legs more often than arms), gait problems, depression or mood impairment, irritability, insomnia, cognitive slowing, forgetfulness, dementia, psychosis, visual disturbances (which may be associated with optic atrophy), peripheral sensory deficits, weakness (that may progress to paraplegia and incontinence), extrapyramidal signs (such as dystonia, dysarthria, and rigidity), and restless legs syndrome. More rarely, individuals may experience skin manifestations like hypo- or hyperpigmentation and bone manifestations like heightened risk of osteoporosis and hip or spine fractures [1].

The diagnosis of pernicious anemia includes analytical findings consistent with macrocytic anemia, characterized by a mean corpuscular volume (MCV) greater than 100 fL and hemoglobin levels below 12 g/dL in women and below 13 g/dL in men [2]. A megaloblastic smear may be found, where macrocytic red blood cells may take an oval shape (macro-ovalocytes). These changes may be accompanied by hypersegmented neutrophils, which consist of neutrophils with six or more segments [5]. These findings can be seen in vitamin B12 and folate deficiencies, as well as with medications that inhibit DNA replication. Autoantibodies directed against intrinsic factor and gastric parietal cell antigens play a role in the pathogenesis of pernicious anemia and serve as important diagnostic tools [3].

Pernicious anemia is also linked to an increased risk of gastrointestinal cancer [6]. Achlorhydria can lead to compensatory hypergastrinemia and cellular metaplasia, as well as promote microbial colonization, resulting in the production of genotoxic byproducts. This association supports the recommendation to perform an initial endoscopy and adopt a lower threshold for evaluating gastrointestinal symptoms in individuals with pernicious anemia [3]. In some cases, there is ineffective erythropoiesis leading to intramedullary hemolysis. This finding may result in elevated bilirubin and lactate dehydrogenase (LDH) levels without reticulocytosis and decreased levels of leukocytes and platelets, indicative of pancytopenia [7,8].

The treatment of pernicious anemia consists of vitamin B12 replacement. This can be administered gradually over several weeks in asymptomatic patients [9]. However, in symptomatic individuals or those presenting with neurological or psychiatric manifestations, replacement therapy should be initiated urgently in order to prevent irreversible neurological deficits [3,10]. Similarly, in cases involving pregnancy or when the patient is a child or an infant, treatment must also be started immediately [3]. Vitamin B12 supplementation can be administered either orally in high doses or parenterally (intramuscularly) [11]. Recent studies have shown no significant difference in the efficacy of raising vitamin B12 levels between oral and intramuscular administration [12,13]. The initial intramuscular dosage is 1000 mcg (1 mg) administered once weekly for a period of four weeks, followed by 1000 mcg monthly for the duration of the patient's life [3]. As for oral administration, the recommended dosage is 1000 to 2000 mcg (1 to 2 mg) daily indefinitely, provided there are no acute anemia symptoms or neuropsychiatric signs [9,11].

## Case presentation

This is the case of a 36-year-old male of African descent. He has no relevant personal medical history, regular medication, or known drug allergies. He is a construction worker and is part of a nuclear family. The patient sought open consultation at the Family Health Unit due to asthenia and fatigue, which had been progressively worsening with increased difficulty in performing work-related tasks over the past three months. He denied any history of blood loss. Concurrently, he reported a sensation of fever (not objectively measured) for the past two days, accompanied by nasal congestion, rhinorrhea, and myalgias. On physical examination, he appeared ill, was afebrile, and was hemodynamically stable, with pale and nonicteric skin and mucous membranes. An episode of vomiting was noted during the consultation. Cardiac and pulmonary auscultation revealed no abnormalities, and abdominal palpation did not reveal organomegaly. Urgent laboratory tests were requested, revealing pancytopenia: hemoglobin of 4.7 g/dL, MCV of 101.9 fL, and mean corpuscular hemoglobin (MCH) of 36.5 pg (macrocytic hyperchromic anemia), where multiple ovalocytes were observed on peripheral blood smear. The laboratory results showed leukocytes of 3.4 x 10^3^/µL with hypersegmented neutrophils, platelets 78 x 10^3^/µL, pronounced anisocytosis, with a reticulocyte count of 1.4% (within the reference interval, indicating normal bone marrow function) and no evidence of iron deficiency.

The patient was immediately referred to the emergency department of the reference hospital, where the diagnostic workup for the underlying pathology was initiated. In the emergency department, two units of red blood cell concentrate were administered.

The patient was admitted to the Intermediate Care Unit of the referral hospital, where he remained for seven days. During hospitalization, laboratory investigations revealed a marked vitamin B12 deficiency (<74 pmol/L), while folate levels were within the reference range (25.3 nmol/L). Elevated indirect bilirubin (1.54 mg/dL), significantly increased lactate dehydrogenase (5273 U/L), and decreased haptoglobin (<10 mg/dL) indicated intravascular hemolysis, although the patient did not present any clinical features indicative of this condition, such as jaundice. A weakly positive direct Coombs test with negative subclasses suggested that a pure hemolytic process was unlikely. Anti-neutrophil cytoplasmic antibodies (ANCA) and antinuclear antibodies (ANA) were negative, excluding autoimmune conditions that may cause anemia of chronic disease. Serum protein electrophoresis revealed no significant abnormalities, excluding multiple myeloma. Anti-parietal cell and intrinsic factor antibodies were strongly positive. An upper gastrointestinal endoscopy revealed mild chronic gastritis, and a neuroendocrine tumor was ruled out. These findings led to the diagnosis of severe pernicious anemia with intramedullary hemolysis.​

Vitamin B12 replacement was initiated in the emergency department via intramuscular injection, administered daily for five days. After two days, serum vitamin B12 levels had been restored (>1476 pmol/L). During hospitalization, following the initial five days of daily intramuscular administration, the patient received weekly injections. At discharge, hemoglobin was 8.0 g/dL, and vitamin B12 levels were corrected. The patient was advised to continue intramuscular cyanocobalamin every two weeks.

Three weeks post-discharge, he resumed work activities and after 10 weeks was asymptomatic with normal hemoglobin levels (14 g/dL), as presented in Table 1. Currently, due to the unavailability of intramuscular vitamin B12, the patient is being treated with oral cyanocobalamin, administered twice daily, and is expected to be continued indefinitely. He is also medicated with omeprazole 20 mg once daily. He remains under follow-up at both the hospital outpatient clinic and the Family Health Unit.

**Table 1:** Laboratory parameters at hospital admission before initiating treatment and after 10 weeks of vitamin B12 replacement therapy. MCV: mean corpuscular volume; MCH: mean corpuscular hemoglobin; LDH: lactate dehydrogenase

Parameter	Normal range	Before treatment	After 10 weeks of treatment
Hemoglobin (g/dL)	13.0–16.5	4.7	14.0
MCV (fL)	80–98	101.9	79.6
MCH (pg)	27.3–33.7	36.5	26.3
Reticulocytes (%)	0.5–2.0	1.4	-
Vitamin B12 (pmol/L)	147.5–590	<74	735
LDH (U/L)	80–225	5273	-
Haptoglobin (mg/dL)	30–200	<10	152
Indirect Bilirubin (mg/dL)	0.1–0.3	1.54	-
Total Bilirubin (mg/dL)	<1.4	2.12	0.37
Platelets (10^3^/uL)	150–450	78	191

The patient provided written informed consent for the publication of this case report.

## Discussion

This clinical case clearly illustrates the importance of the family physician as a clinician who provides longitudinal medical follow-up and understands the patient within their context. The identification of vague yet persistent symptoms was critical in diagnosing a condition that, although not rare, can remain silent for years, and when it does present clinically, it is often in an advanced stage.

Pernicious anemia is an autoimmune disease that interferes with vitamin B12 absorption, and its slow progression may hinder early diagnosis. In the case described, the patient already exhibited laboratory criteria for severe anemia with signs of intravascular hemolysis, despite the absence of any neurological symptoms [14,15]. It is important to highlight that, despite the laboratory severity, the clinical presentation was subtle and non-specific, an occurrence that is not uncommon in the context of longitudinal care and one that underscores the importance of continuous monitoring and active listening.

Early identification of vitamin B12 deficiency is important to prevent potential complications, such as the risk of developing gastric cancer [2]. It could be argued that an earlier vitamin B12 assessment might have expedited the diagnosis. However, in addition to the patient not adhering to the recommended follow-up care at the Family Health Unit, up until the date of the consultation described in the case, the patient did not present any risk factors for vitamin B12 deficiency, such as gastric or small intestine resections, inflammatory bowel disease, use of metformin for more than four months, use of proton pump inhibitors or histamine H2 blockers for more than 12 months, veganism or strict vegetarianism, or age over 75 years old [16]. Similarly, no suggestive complaints or family history would have warranted further investigation. Therefore, the absence of a prior request for this laboratory parameter appears clinically justified.

According to the NICE (National Institute for Health and Care Excellence) guidelines, vitamin B12 deficiency should be considered in patients presenting with macrocytic anemia, neurological symptoms, or relevant risk factors, as mentioned above. Initial evaluation involves measuring serum B12 levels, and if results are borderline, further testing, such as methylmalonic acid, homocysteine, or intrinsic factor antibodies, may be appropriate [17].

The exclusion of malignant gastric pathology, namely gastric adenocarcinoma or neuroendocrine tumor, was likewise an essential step, given the increased risk associated with pernicious anemia [6]. Upper gastrointestinal endoscopy with appropriate histological assessment enabled the exclusion of these conditions, thereby contributing to a clearer etiological understanding and facilitating safe therapeutic planning.

The clinical and laboratory response to therapy was rapid and complete following the initiation of vitamin B12 replacement. This finding underscores the potential reversibility of most clinical and hematological manifestations when the diagnosis is made in a timely manner [18]. The treatment of pernicious anemia is simple, safe, and accessible, typically involving daily oral or biweekly intramuscular vitamin B12 administration [12]. In this particular case, the patient started reposition treatment with intramuscular vitamin B12 administration, but after hospital discharge, oral vitamin B12 was prescribed due to the unavailability of the injection for medical prescription and collection at the pharmacy at that time. However, it is a chronic therapy that requires regular monitoring and long-term adherence. Follow-up in primary care ensures appropriate clinical and laboratory monitoring, helps prevent relapses, and allows for the management of any associated complications.

In summary, this case highlights the importance of continuity of care, attention to non-specific signs, and the integration of clinical investigation with a holistic understanding of the patient, which are the core competencies of General and Family Medicine.

## Conclusions

This case reinforces the importance of continuity in the doctor-patient relationship, which facilitates the detection of subtle changes in health, even when symptoms are nonspecific. A patient-centered approach, combined with the knowledge accumulated over time, enabled the effective identification and treatment of a clinical situation with the potential for severe progression.

Pernicious anemia, although a straightforward condition to treat, can go unnoticed for years and only present in advanced stages. The absence of neurological symptoms does not exclude the presence of a significant vitamin B12 deficiency, as demonstrated in this case, where severe anemia and intravascular hemolysis coexisted. Timely diagnosis and appropriate treatment enabled a rapid clinical and laboratory recovery, highlighting the impact that early intervention can have on prognosis. Regular follow-up in primary care ensures adherence to therapy and provides the necessary monitoring to prevent complications, thereby safeguarding the patient's quality of life. Cases like this underscore the value of General and Family Medicine in the detection and management of chronic diseases, even when they present in a silent or atypical manner.
